# Visible Enamel Defects in Adolescents: How Do their Peers View Them?

**DOI:** 10.5005/jp-journals-10005-1561

**Published:** 2018

**Authors:** Ullal A Nayak, Apurva Pawar, Damodhar Kappadi, Deepesh Prajapati, Kangkan J Roy, Saakshe Wadhwa

**Affiliations:** 1 Department of Preventive Dental Science, Faculty of Dentistry, IBN Sina National College for Medical Studies, Jeddah, Kingdom of Saudi Arabia; 2-6 Department of Pedodontics and Preventive Dentistry, NIMS Dental College, Jaipur, Rajasthan, India

**Keywords:** Dental appearance, Enamel defects, Judgements

## Abstract

**Aim:**

The present study was aimed to determine whether adolescents make social judgments about other children who have noticeable enamel imperfections.

**Materials and methods:**

Two schools with very different socio-economic profiles were selected using a stratified random sampling method. Children aged 13–14 years (school class 9) and 15–16 (school class 11) were randomly selected from these schools totaling to a sample size of 200. Half the participants rated full-face photographs of a boy and girl without an enamel defect, and the other half-rated the same two photographs with the digitally modified incisors. The photographic subjects were rated on a four-point Likert's scale consisting of six positive and five negative descriptors.

**Results:**

Based on the gender of the photographic subject, the mean total attribute score was almost identical. However, it was lower for photographic subjects showing enamel defects as compared to those without.

**Conclusion:**

The dental appearance can influence value judgments in children.

**Clinical significance:**

It is important to treat the enamel lesions and various developmental defects of the enamel as it may lead to a negative social judgment by peers.

**How to cite this article:**

Nayak UA, Pawar A, Kappadi D, Prajapati D, Roy KJ, Wadhwa S. Visible Enamel Defects in Adolescents: How Do their Peers View Them?. Int J Clin Pediatr Dent, 2018;11(6):479-482.

## INTRODUCTION

Many times we are judged by our appearance, including dento-facial esthetics. Physical appearance influences how people feel about themselves, as well as it impacts how they are judged by others. A study done by Dion in 1970 infers that “attractive” kids probably escape the offenses more than “unattractive” kids and that “unattractive” kids are seen as being naughtier or dishonest.^1^ Further, studies were done by Shaw and co-workers also supports this hypothesis in which they used digitally changed photos of subjects with an assortment of malocclusions and the participants rated the subject's photos for various individual traits.^2^ Higher ratings are reported for social class, friendliness, popularity, and intelligence which are obtained for photographs showing ideal incisor relationships. Photos received the highest scores for compliance and honesty when the incisors were prominent whereas most aggressive ratings were given when an incisor was missing.^3^

The influence of dental caries, as well as tooth discoloration on social judgments, have been investigated in the past.^4^ Further, a sound dentition is often related to an intellectual and skilled, in contrast to individuals presenting with caries on anterior teeth.^5^

Various conditions inherited or acquired may alter the appearance of teeth from their acknowledged standards, such as enamel defects, dental trauma, and morphological defects. Although personality traits are observed to be seen based on the dental appearances, there is insufficient information regarding the judgment of children or adolescents related to their dental status.^6^

Along these lines, the key factor inciting this study was to get information regarding clinical interventions in adolescents displaying visible enamel defects. Thus this study aimed to determine whether adolescents make social judgments regarding other children who present with noticeable enamel imperfections. The objective of the study was to assess the role of age, gender or socio-economic status on how adolescents appraise other children with or without enamel defects.

## MATERIALS AND METHODS

Two schools which are socio-economically different were selected using a stratified random sampling method. School A, an urban school in Jaipur, Rajasthan had high educational attainment and was situated in a relatively affluent area. School B was a small rural school in Kant. A total of 200 adolescents were selected for the study (100 from each school). Children aged 13–14 years (class 9) and 15–16 years (class 11) were randomly selected from these schools. The study nature and methodology was explained to the parents and their written consent was obtained before its beginning.

A questionnaire was formulated to measure their value judgments in relation to dental appearance, and its face, content and construct validities were assessed. This social attribute questionnaire was based on a format of 4-point Likert scale where the adolescents rated 11 attributes which had six positive and five negative attributes (Table 1). The participant's responses for each attribute were added to derive the total attribute score (TAS). The positive attributes scored as 4, 3, 2 or 1 based on how strongly the adolescent had associated the virtue with the adolescent on the photograph. For negative attributes, the scoring was reversed. Thus, the TAS ranged from 11 being most negative to 44 being most positive.

**Fig. 1:**
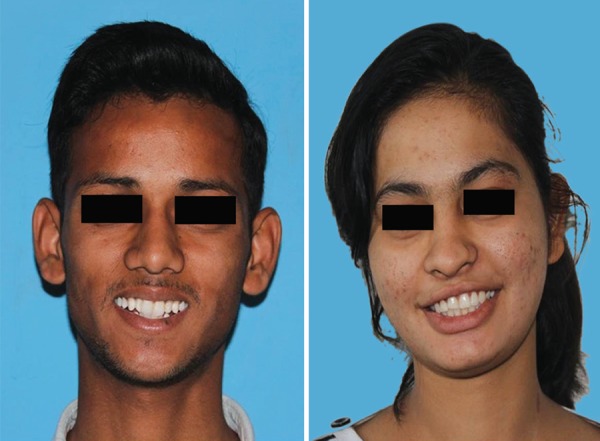
Photographs of 15-year-old boy and girl without enamel defects on anterior teeth

**Fig. 2:**
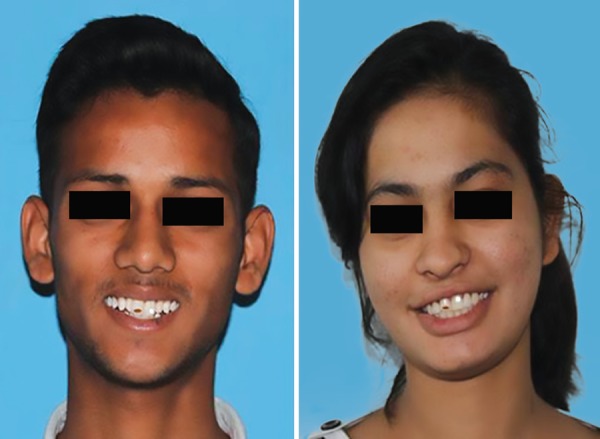
Photographs of 15-year-old boy and girl with digitally superimposed enamel defects on anterior teeth

Two photographs, that were to be rated, were a color digital photograph displaying full face of a 15-year-old boy and a girl who presented with good oral health (Fig. 1). Onto the photographic subject's upper right central incisor, a brown enamel defect (well-localized) was then digitally superimposed, and a white opacity was painted on the upper left incisor (Fig. 2).

The questionnaire packs on the data collection day were randomly distributed to 50 students each from classes 9 and 11. Each class show the photographs of adolescents either with or without enamel defects. Thus, the participants were equally distributed and the pupils of different classes never collided. Thus, they were unaware of being judged by digitally modified photographs of the same adolescents.

## RESULTS

All the data collected were statistically analyzed using SPSS version 19. Table 2 shows the mean total attribute scores (TAS) for photographic adolescents according to school, age, and gender. It was seen that the lowest TAS, i.e., the most negative evaluation was 23.67 which was rated by class 11 boys at school A after viewing female photographic adolescents with enamel defects. The highest TAS, i.e., the most positive evaluation was 34.18 which were rated by class 11 boys at school B after viewing male photographic subject without enamel defects.

The mean TAS for male and female photographic adolescents, with and without enamel defects was seen in Table 3. The mean TAS recorded was almost identical on the basis of the gender of the photographic adolescents. However, the mean TAS for photographic adolescents with enamel defects was lower than those without enamel defects. Table 4 shows the comparison of mean TAS score for photographic adolescents with enamel defects based on gender, school and age groups using independent sample t-test (*p* <0.05). The only significant difference was seen between school A and school B students for male photographic adolescents.

**Table 1:** Social attributes questionnaire

*Question*	*Definitely no*	*No*	*Yes*	*Definitely yes*
Is he/she kind?				
Is he/she honest?				
Is he/she naughty?				
Is he/she clever?				
Is he/she rude?				
Does he/she care about his/her appearance?				
Is he/she careful?				
Is he/she lazy?				
Is he/she confident?				
Is he/she helpful?				
Is he/she stupid?				

**Table 2:** Mean total attribute scores (TAS) for male and female photographic adolescents with and without enamel defects

*School*	*Class*	*No of adolescents*	*TAS of photographic adolescent without enamel defect Mean ± SD*		*No of adolescents*	*TAS of photographic adolescent with enamel defect Mean ± SD*	
			*Boy*	*Girl*		*Boy*	*Girl*
School A (Urban)	Class 9 boys	17	29.29 ± 3.361	29.59 ± 2.829	11	26.09 ± 3.448	28.36 ± 2.873
	Class 9 girls	8	26.38 ± 4.34	27.75 ± 0.886	16	26.75 ± 2.594	28.56 ± 3.577
	Class 11 boys	10	28 ± 3.887	29 ± 2.261	6	25.16 ± 3.71	23.67 ± 3.585
	Class 11 girls	15	29.73 ± 3.127	30.27 ± 4.113	21	26.38 ± 3.089	26.57 ± 3.076
School B (Rural)	Class 9 boys	16	32.5 ± 3.112	32.44 ± 4.33	19	28.42 ± 3.237	27.26 ± 3.955
	Class 9 girls	9	31.11 ± 5.395	31.33 ± 3.905	6	28.67 ± 3.141	29.5 ± 2.738
	Class 11 boys	16	34.18 ± 3.655	33.06 ± 4.024	19	27.05 ± 4.636	29.211 ± 4.661
	Class 11 girls	9	30.55 ± 3.282	29.22 ± 3.961	9	28.22 ± 4.79	30 ± 4.358

**Table 3:** Mean total attribute score (TAS) for each photographic adolescents

*Photographic adolescent*	*Total students*	*Mean TAS*
Boy without enamel defects	100	30.56
Boy with enamel defects	107	27.1
Girl without enamel defects	100	30.62
Girl with enamel defects	107	27.93

## DISCUSSION

Being attractive has been a momentous benefit for both children and adults in almost every sphere of judgment, treatment, and behavior. For appearance to have any reliable impact on differential judgment or treatment, persons must agree about who is attractive and who is not; and they ought to bring out differential expectations from others.^7^ Hence, this evaluated how adolescents appraise their peers with or without enamel defects. The study was conducted in urban and rural schools of the city, by which information can be obtained by children from different social class.

The study confirms that adolescents do make value judgments about other children based on enamel imperfections. This supports several previous studies which also confirm that negative social judgments are made when the dental appearance differs from expected norms.^8,9^ The longing for a better esthetic appearance is driven by psychological factors and their desire for a perfect smile.^10^

This study also found adolescent boys or girls of the same group made similar social judgments. These findings, however, differ from a study done by Craig et al. which showed that girls were more positive in their judgments as compared to their male peers in relation to visible enamel defects.^11^

The age of the student did not appear to have any effect on their judgment. The 13–14-year-old made similar social judgments in relation to enamel imperfections as the 15–16-year-old. This was in accordance with the study done by Rodd et al. in which they found that a child's age did not correlate well with their social judgment made in the context of visible incisor trauma.^12^ However, the findings of Patel et al. are not in consensus with this fact. They found that school class 10 students gave a more negative rating than the school class 7 students.^8^ The literature gives various reasons for these different types in judgments, and older children may have themselves come across or are aware of the occurrence of such enamel defects in them or among their peers and adolescents show maximum self-monitoring behavior which makes them conscious and emotional thereby increasing the need of cognitive efforts to be socially acceptable.^12^

It was postulated that children belonging to the lower socioeconomic group are associated with poorer dental health and lower treatment expectations. Hence, they are likely to be more positive and generous in their judgment for the children with enamel defects. The findings of this study supported the same. A relatively significant difference was found between the social judgments made by the school A and school B students concerning the photographic subject with enamel imperfections. Whereas study done by Craig et al. who evaluated in a similar manner founded that the socioeconomic status did not affect children's views.^11^ Similarly, a study conducted by Kershaw et al. showed that sociodemographic characteristics of participant had no relation on the personality, but overall negative judgments were recorded for the visible dental defects when compared to normal teeth.^9^

**Table 4:** Comparison of mean TAS score for photographic adolescents with enamel defects among gender, school and age groups

*Evaluation of male photographic subject with enamel defects*					
*Variables*		*No. of participants*	*Mean TAS score*	*SD*	*p value*
Gender of participants	Male	55	27.13	3.921	0.943
	Female	52	27.08	3.325	
School of participants	School A (Urban)	54	26.30	3.045	0.019
	School B (Rural)	53	27.92	3.999	
Age group of participants	Class 9	52	27.44	3.171	0.349
	Class 11	55	26.78	4.012	
*Evaluation of female photographic subject with enamel defects*					
*Variables*		*No. of participants*	*Mean TAS score*	*SD*	*p value*
Gender of participants	Male	55	27.764	4.1986	0.651
	Female	52	28.115	3.7971	
School of participants	School A (Urban)	54	27.204	3.6517	0.056
	School B (Rural)	53	28.679	4.2189	
Age group of participants	Class 9	52	28.154	3.4944	0.583
	Class 11	55	27.727	4.4366	

*p* ≤ 0.05 is significant

## CONCLUSION

The conclusions of this questionnaire study include:

- Dental appearance influences adolescents in making value judgments.
- Age and gender was not a significant predictor of how adolescents viewed their peers with enamel defects.
- Socioeconomic status does affect the judgment of adolescents.

## CLINICAL SIGNIFICANCE

Based on the findings of this study, we can infer that adolescents make negative social judgments about peers with visible enamel defects. A wide range of enamel defects are commonly seen including white enamel opacities, and it is important to report and treat these lesions early to avoid its negative implications.
